# Supplementary material for: Health-related quality of life of the Vietnamese during the COVID-19 pandemic
Source: PLoS One. 2020 Dec 18;15(12):e0244170. doi: 10.1371/journal.pone.0244170 (PMC7748152; doi:10.1371/journal.pone.0244170)
Supplement: Attachment — Submitted filename: 11Nov_Response to Reviewers.pdf [file pone.0244170.s002.pdf]

RESPONSE TO JOURNAL REQUIREMENTS:

1. Please ensure that your manuscript meets PLOS ONE's style requirements.

Response: We have consulted the instruction on the format for PLOS ONE journal, and adjusted our manuscript accordingly.

2. Ethical considerations

a. Please provide additional details regarding participant consent

b. Once you have amended this/these statement(s) in the Methods section of the manuscript, please add the same text to the "Ethics Statement" field of the submission form (via "Edit Submission").

Response: We have added "Ethical considerations" in the Methods section (line 157-163) as follow:

"The beginning of the survey provided general information of the study, and clearly stated that as an individual proceeded with answering the questions, he or she would have given informed consent to participate in the study and understood the right to withdraw this consent at any time by simply exiting the survey Google Form. The study design was approved by the Ethical Review Board for Biomedical Research at Hanoi University of Public Health (Identification number: 144/2020/YTCC-HD3)."

3. Please include your tables as part of your main manuscript and remove the individual files. Please note that supplementary tables should be uploaded as separate "supporting information" files.

Response: We revised our manuscript accordingly.

4. Your ethics statement should only appear in the Methods section of your manuscript.

Response: We have moved the "Ethical considerations" to Method section.

RESPONSE TO REVIEWERS' COMMENTS

1. Is the manuscript technically sound, and do the data support the conclusions?

Reviewer #1: Yes *Reviewer #2: Partly*

2. Has the statistical analysis been performed appropriately and rigorously?

Reviewer #1: Yes *Reviewer #2: Yes*

3. Have the authors made all data underlying the findings in their manuscript fully available?

Reviewer #1: No *Reviewer #2: Yes*

Response: We have added a section "Data availability" for further request at the end of our revised manuscript (line 348-345).

4. Is the manuscript presented in an intelligible fashion and written in standard English?

Reviewer #1: Yes *Reviewer #2: Yes*

Review #1 Comments

1. **General Comments:** The manuscript contributes to body of scientific knowledge by providing insight into the living condition and health status of the Vietnamese population during COVID quarantine measure. However, the sample size is rather low for generalization to a population of over 90 Million. Although the authors expressed this as one of the limitations of the study, rather a sample size of 10 for the Government quarantine facility is a major flaw to the study.

Response:

During the period of 01 April 2020 to 30 May 2020, Vietnam had about of 4,000 - 18,000 people experienced a 14-day quarantine at a Government facility; about 73,923 (at peak) - 2000 people experienced a 14-day self-isolation at their private places and the rest practiced physical distancing.

We agree with the reviewer that our sample size was very small. However, due to the fact that this study was self-funded by the research team, we unfortunately did not have strong resources to promote our online survey widely. In addition, this study was to give a snapshot of the impacts of the Vietnamese government' intensive response to COVID-19 on HRQOL of the general population during the pandemic's peak. Regardless of small sample size, findings of this study would still benefit our government and individuals in following our government' guidance to prevent and control COVID-19.

For the very small sample of 10 responses from whom being quarantined in a Government quarantine facility at the time, it could be explained as follow: Since the Internet inside Government quarantine facilities was not stable/available, there was a huge barrier in collecting data of this group through an online survey. In fact, we even did not expect any response from this group. It was quite fortunate that,

in the end, we somehow got 10 respondents, so our team decided to respect the data and clearly acknowledge the study' limitations.

Introduction:

2. The syntax errors in lines 40 and line 57 should be corrected. prepositions are missing

Response: We have these errors fixed as follow:

Line 40: *"Literatures about COVID-19's negative impacts on the health-related quality of life among people with underlying medical condition(s) were also proven elsewhere"* (see line 43-45)

Line 57: *"It can be hypothesized that, strict mobility restrictions, difficulties in usual and daily activities, along with negative physical and psychological impacts resulted from quarantine or physical distancing, may lead to lower quality of life."* (see line 65-68)

3. Literature review lacked HRQOL among the Vietnamese population pre-COVID. The authors should include a few sentences on this.

Response: We agree with the reviewer and revised the text accordingly (see line 58-63)

"*A study in 2017 on HRQOL of the Vietnamese, however applying Chinese' preferences, reported a mean EQ-5D-5L value of 0.91 and EQ-VAS score of 87.4 [21]. In the earlier this year, study on HRQOL reference data using Vietnamese EQ-5D-5L showed a slightly lower HRQOL of the Vietnamese at 80.10 and 0.94 for EQ-VAS and EQ-5D-5L value respectively [22]. Considering such findings belong to pre-COVID period, the people HRQOL perhaps would show a decline in the pandemic time."*

Methods:

4. Line 82-83: How does ending data collection process at the end of strict lock-down prevent information bias?

Response: Thank you for the comment to help us further clarify our point. This is because we went for a cross-sectional study design to measure the HRQOL of Vietnamese people when the COVID-19 pandemic was at its (first) peak in Vietnam. Therefore, extending the data collection pass the strict lock-down period could have our findings being prone to recall bias, which hence information bias.

5. Line 83: Google survey tool should be properly described and reference

Response: The "Google survey tool" was named under "Google Forms" so we revised this as follow:

"Data was collected online based using Google Forms, and participants were invited to self-answer the online survey. Google Forms is a survey administration software that allows collecting information

from respondents through surveys. The collected information can be automatically entered into a spreadsheet for data extraction [24].” (line 105-109)

6. Line 87: Use of "e.g" and "etc" should be discouraged in biomedical writing

Response: This issue was addressed accordingly.

7. Line 90-93: The use of sort questions to classify participants into the three groups is unnecessary, reason being that the grouping in this study can only be objectively informed by the living condition during the quarantine policy rather their COVID status.

Response: Thank you for the comment. In fact, with thousands of cases that need to be assigned for being “self-isolation at private place” by the MOH in such a short time, a delay in informing about these individuals’ F2-4 status was expected. Hence, the research team applied sorting questions to detect potential F2-4 cases. We have revised this part as follow (line 120-129):

“Participants were asked to claim whether they were currently in government quarantine facilities (F1) or in isolated residence areas (F2-3-4). It was assumed that there could be of “self-isolated at private places” cases (F2-3-4), whom had not yet assigned by the MOH for strict quarantine, and was not sure about their F2-3-4 status. Therefore, sorting questions were applied to determine whether the respondent: (1) shared either living residence or workplace or travel group with the suspected cases; (2) were in the same row or had a seat in two rows from the suspected cases in public transport; and/or (3) had face-to-face conversation with the suspected cases within a distance of less than two meters. Participants, who reported having any of these three experiences, were categorized into the “self-isolation at private place” group.”

8. Line 95-102: Can compliance to the quarantine policy be a major confounder in this study?

Response:

We thank the reviewer for your suggestion. In this study, our main purpose is to compare the HRQOL across participant's characteristics, stratified 3 groups. To serve this purpose the compliance variable was used as the main predictor (i.e., we compared the difference in HRQOL among compliance groups). Regarding the potential confounding effect of the compliance variable. We consider it is not a confounder, in both theory and statistical aspects:

In the theory aspect:

A confounder is defined as “a factor that is related to the risk factor of interest and, independently of this, to the outcome, but is not an intermediate factor on the causal path between the risk factor and outcome” [1]. In our study, the compliance variable appears to be a mediator, not a confounder (i.e., age, sex, education, occupation, etc may lead to the difference in compliance status, and in turn, compliance status potentially affects the HRQOL).

In the statistical aspect:

As shown in Table 2 and Table 3, there is no significant difference in HRQOL among compliance groups, thus compliance might not be considered as a confounder.

[1] Rothman, K., Greenland, S., & Lash, TL. (2008). Modern Epidemiology, 3rd Edition. Lippincott Williams & Wilkins.

9. Line 106 -108: These statements require proper references

   Response: We added references (# 25 -28) accordingly.

10. Line 120-121 Analysis: The assumptions for applying Mann-Whitney U and Kruskal Wallis tests should be clearly stated in addition to their limitations. No statement on significance level was made. This is a vital to the study

Response:

Thank you for your comment. We have added the text regarding the assumptions for applying Mann-Whitney U and Kruskal Wallis tests, and provide the significance level used in this paper:

"Since the sample size of the two groups: "In Government quarantine facilities" and "Self-isolation at private place" were small, and the score of EQ-5D-5L and EQ-VAS were not normally distributed, the Mann–Whitney U tests and Kruskal Wallis tests were carried out to identify the difference in HRQOL among participants." (line 145-149)

And

"A significance level of 0.05 was used for all statistical tests." (line 154-155)

Results: In general, the authors should clearly state significant findings in this study

11. Table 1: Shows a very young population (31.8 years). Could this have confounding influence on HRQOL and quarantine? This worth exploring in the analysis by controlling for the age. This was corroborated with an interesting finding in Table 2 where 44+ years have reduced HRQOL compared to younger age groups

Response:

We appreciate your comment. Although the EQ-5D-5L scores were different between age groups among those self-isolated at private places (Table 3), age might not have a confounding effect on quarantine to HRQOL. The quarantine status was based on their contact history and the government guideline, so that age and quarantine status were literally not related.

We have discussed more on the point that the average HRQOL of young people is higher than the average HRQOL of the general population. (line 270-280)

12. Table 2: With only 10 participants in (F1 group) the group that was hypothesized to have worse HRQOL, how reliable is this? Furthermore, the Kruskal Wallis test also show too many empty cells and therefore make some of the analysis unreliable

Response: We appreciate your comment. This study was conducted in the very early stage of the COVID-19 pandemic in Vietnam so that the sample size of the group in government quarantine facilities was small (10 participants). Thus, there was the limited power of the statistical tests, we have acknowledged this as a limitation of this study. Please see the limitations (line 322-325)

13. Tables 2 & 3: Kruskal Wallis test is an Omnibus test and therefore require a Poc hoc analysis to reflect the significant combinations. The authors should include this in their analysis.

Response: We agree with the reviewer, we added po-hoc analysis for any statistically significant results from Kruscal Wallis tests in table 2 and 3 and in the text line 149-150

"The Dunn's tests were used as the Poc-hoc analysis of the Kruskal-Wallis to examine the differences among multiple pairwise comparisons".

Discussion:

14. Line 191-194: The main findings of HRQOL among the three groups studied were statistically insignificant from the results reported but the author based the main conclusion on these findings. It will be desirable if the authors can include other salient significant findings from the inferential statistics in both the discussion and the summary (e.g Compliance, age, sex, chronic illness). Critically appraisal of these factors will give more credence to the study

Response: Thank you for the comment. We revised the discussion accordingly (see line 255-263)

"This study has provided the pattern of the HRQOL for the Vietnamese general population during the COVID-19 pandemic. Differences in HRQOL among the general population not needing compulsory isolation, people in government quarantine facilities, and people implemented self-isolation at private places were given. For the three groups of isolation status, "do not need isolation"; "currently in government quarantine facility" and "self-isolation at private place", the means of EQ-VAS scores reported at 88.54, 90.50, 86.54; and the means of EQ-5D-5L values were 0.95, 0.94, 0.93, respectively. Overall, HRQOL were higher in younger people, and in people with better health and incomes. The percentages of people having full health were higher than 50% among all three groups of different isolation status."

15. References: All the internet references lacked accessed date. This should be indicated in the relevant

Response: Thank you for the comment. The accessed date is linked with *"[cited (date) 2020]"* in our Reference section.

Reviewer #2 Comments

1. Limitation of the sample size has been addressed in your discussions, however the composition of your sample and its effect on your final outcome may require further discussion/clarification owing to the association of higher HRQOL with higher education levels [I, II] and 92.3% of your respondents having university degrees and above.

References

I. Hoi, Le & Chuc, Nguyen & Lindholm, Lars. (2010). Health-related quality of life, and its determinants, among older people in rural Vietnam. BMC public health. 10. 549. 10.1186/1471-2458-10-549.

II. Mielck, Andreas ,Reitmeir, Peter, Vogelmann, Martin, Leidl, Reiner(2012)Impact of educational level on health-related quality of life (HRQL): results from Germany based on the EuroQol 5D (EQ-5D) European Journal of Public Health.DO 10.1093/eurpub/ckr206

III. Gil-Lacruz, M., Gil-Lacruz, A.I. & Gracia-Pérez, M.L. Health-related quality of life in young people: the importance of education. Health Qual Life Outcomes 18, 187 (2020). https://doi.org/10.1186/s12955-020-01446-5

Response:

We agree with the reviewer. We clearly stated the bias in Discussion (see line 318-322)

"*First, the data was collected based on an online survey which could be prone to selection bias. Due to the nature of accessing internet and technology was, in fact, less common for the elderly and ones with low socio-economic status, most of our participants were young and from the middle class. The elderly, poor and other vulnerable groups did not seem to appear in our sample, and so their HRQOL status was not able to be recorded".*

Other minor issues include:

2. Line 138: Data set incorrectly ordered for the dimension character listed prior and its corresponding numerical value. The correct order should be 297, 259, and 206 respectively.

Response: We have adjusted this accordingly for the Results section, line 186

3. Line 166: Table 2 should read mean and standard deviation

Response: We have adjusted this accordingly for the Results section, line 220

4. Line 161-163 report of result may require restructuring of sentence for clarity.

Response: We have revised this part as follow:

"Having problems with usual activities and having pain/discomfort were not reported among people in the government quarantine group. However, these dimensions were both reported to be lower at 6.5% among people do not need to isolation, yet were respectively higher at 8.8% and 14.0% among people in the self-isolation group. (See line 214-218)
